# Genetic Suppression of Lethal Mutations in Fatty Acid Biosynthesis Mediated by a Secondary Lipid Synthase

**DOI:** 10.1128/AEM.00035-21

**Published:** 2021-05-26

**Authors:** Marco N. Allemann, Eric E. Allen

**Affiliations:** a Marine Biology Research Division, Scripps Institution of Oceanography, University of California, San Diego, La Jolla, California, USA; b Center for Marine Biotechnology and Biomedicine, Scripps Institution of Oceanography, University of California, San Diego, La Jolla, California, USA; c Division of Biological Sciences, University of California, San Diego, La Jolla, California, USA; Kyoto University

**Keywords:** deep sea, lipids, *Photobacterium*, high pressure, polyunsaturated fatty acid

## Abstract

The biosynthesis and incorporation of polyunsaturated fatty acids into phospholipid membranes are unique features of certain marine *Gammaproteobacteria* inhabiting high-pressure and/or low-temperature environments. In these bacteria, monounsaturated and saturated fatty acids are produced via the classical dissociated type II fatty acid synthase mechanism, while omega-3 polyunsaturated fatty acids such as eicosapentaenoic acid (EPA; 20:5*n-*3) and docosahexaenoic acid (DHA; 22:6*n-*3) are produced by a hybrid polyketide/fatty acid synthase—encoded by the *pfa* genes—also referred to as the secondary lipid synthase mechanism. In this work, phenotypes associated with partial or complete loss of monounsaturated biosynthesis are shown to be compensated for by severalfold increased production of polyunsaturated fatty acids in the model marine bacterium Photobacterium profundum SS9. One route to suppression of these phenotypes could be achieved by transposition of insertion sequences within or upstream of the *fabD* coding sequence, which encodes malonyl coenzyme A (malonyl-CoA) acyl carrier protein transacylase. Genetic experiments in this strain indicated that *fabD* is not an essential gene, yet mutations in *fabD* and *pfaA* are synthetically lethal. Based on these results, we speculated that the malonyl-CoA transacylase domain within PfaA compensates for loss of FabD activity. Heterologous expression of either *pfaABCD* from *P. profundum* SS9 or *pfaABCDE* from Shewanella pealeana in Escherichia coli complemented the loss of the chromosomal copy of *fabD in vivo*. The co-occurrence of independent, yet compensatory, fatty acid biosynthetic pathways in selected marine bacteria may provide genetic redundancy to optimize fitness under extreme conditions.

**IMPORTANCE** A defining trait among many cultured piezophilic and/or psychrophilic marine *Gammaproteobacteria* is the incorporation of both monounsaturated and polyunsaturated fatty acids into membrane phospholipids. The biosynthesis of these different classes of fatty acid molecules is linked to two genetically distinct co-occurring pathways that utilize the same pool of intracellular precursors. Using a genetic approach, new insights into the interactions between these two biosynthetic pathways have been gained. Specifically, core fatty acid biosynthesis genes previously thought to be essential were found to be nonessential in strains harboring both pathways due to functional overlap between the two pathways. These results provide new routes to genetically optimize long-chain omega-3 polyunsaturated fatty acid biosynthesis in bacteria and reveal a possible ecological role for maintaining multiple pathways for lipid synthesis in a single bacterium.

## INTRODUCTION

Selected lineages of marine *Gammaproteobacteria*, predominantly from deep-sea and/or low-temperature ocean habitats, contain a specialized genetic pathway for the *de novo* biosynthesis of long-chain omega-3 polyunsaturated fatty acids (PUFA), specifically eicosapentaenoic (EPA; 20:5*n-*3) and docosahexaenoic (DHA; 22:6*n-*3) acids (1, 2). The genes responsible for their biosynthesis, designated *pfaABCDE*, have been well characterized in terms of their environmental and phylogenetic distribution and, more recently, at the level of enzymatic activity (3–5). Typically, these strains produce either EPA or DHA as a small percentage of total fatty acids, i.e., approximately 5 to 10% of total cellular content (1, 6, 7), with the majority comprised of saturated (SFA) and monounsaturated (MUFA) species. Numerous studies have demonstrated the increased incorporation of omega-3 PUFA into membrane phospholipids in response to culturing parameters that elicit membrane gelling effects, such as increased hydrostatic pressure and/or low temperature (7–12).

**FIG 1:**
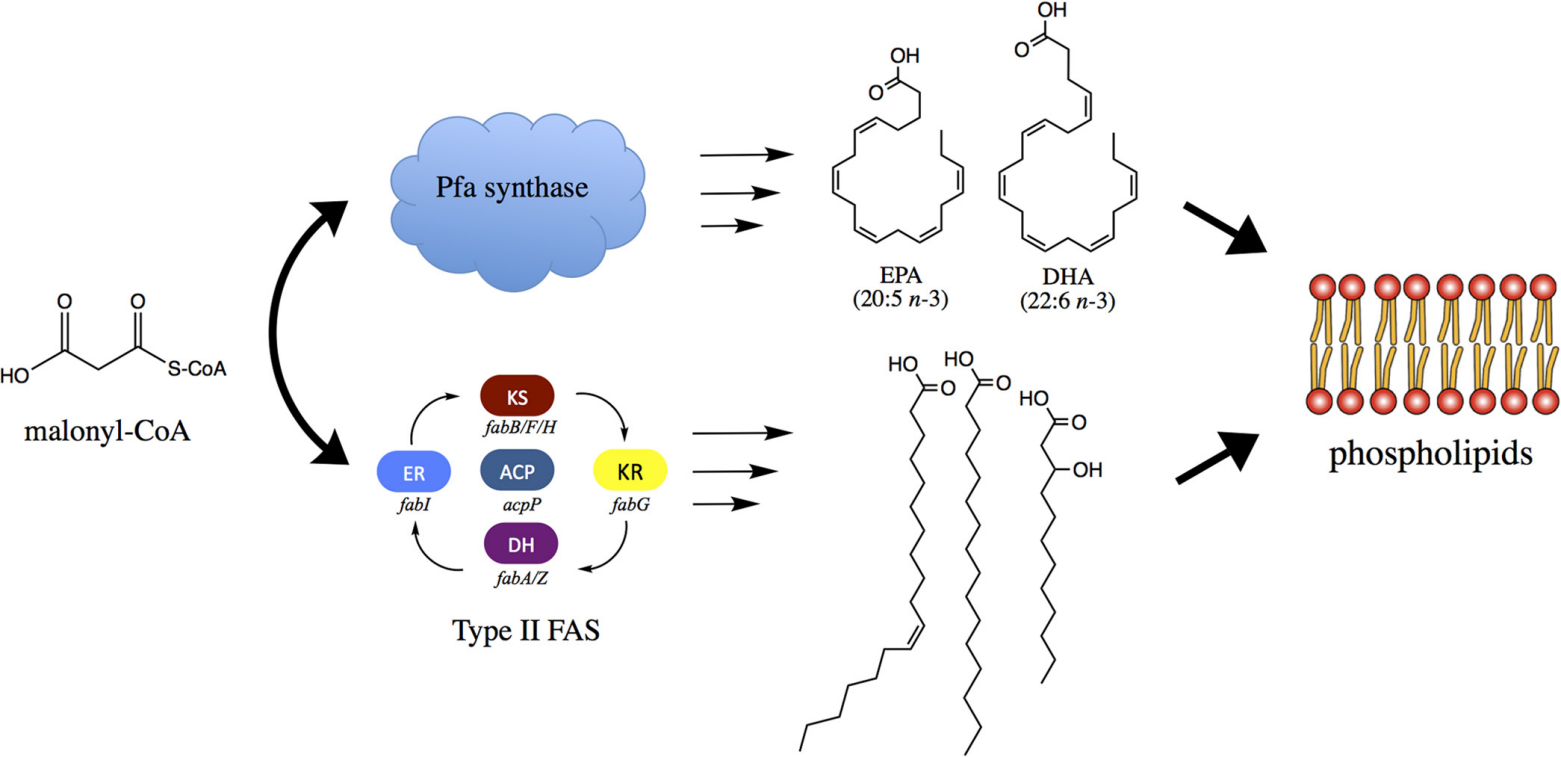
Model for interaction of Pfa synthase and type II FAS in *P. profundum* SS9. Both the Pfa synthase and type II FAS utilize malonyl-CoA for production of their respective products. End products of both pathways are incorporated into phospholipid membranes.

To date, all known bacterial strains that synthesize omega-3 PUFA via the Pfa synthase, or “secondary lipid,” pathway also retain the highly conserved type II fatty acid synthase (FAS) found throughout the domain *Bacteria* and well studied in Escherichia coli (13–15). As shown in Fig. 1, the type II FAS produces SFA and MUFA products and utilizes the same malonyl-CoA precursors as the Pfa synthase (3, 13, 16), with the acyl products of both pathways destined for incorporation into membrane phospholipid (17–19). Each pathway retains the full complement of enzyme activities required for the biosynthesis of fatty acid products and are encoded either by discrete genes in the type II FAS or by embedded enzymatic domains within the multidomain Pfa enzyme complex (see Fig. S1 in supplemental material). How these independent fatty acid biosynthesis pathways and their complementary genetic components interact to maintain membrane homeostasis in omega-3 PUFA-producing marine bacteria has not been thoroughly investigated.

Given the presence of two discrete pathways for the biosynthesis of unsaturated fatty acids, we hypothesized that genes critical for MUFA synthesis could be disrupted in the native producing organism and compensated for by PUFA produced by the Pfa synthase. Previous work utilizing the fatty acid biosynthesis inhibitor cerulenin (7, 20) or genetic disruption of the *fabF* gene, which encodes the key condensing enzyme, β-ketoacyl-ACP (acyl carrier protein) synthase II, involved in 18:1 production (9), showed that compensatory increases in PUFA content mitigated the loss of MUFA biosynthesis in the marine bacterium Photobacterium profundum SS9. Additionally, transposon screening for pressure-sensitive mutants of *P. profundum* SS9 revealed that *fabB* was not an essential gene and that its disruption led to a pressure-sensitive phenotype (21). Taken together, these results indicate that the products of either pathway could satisfy the cellular demand for unsaturated phospholipids.

To gain greater insight into the interaction of these two biosynthetic pathways, we engineered mutants of *P. profundum* SS9 with various levels of MUFA-deficient phenotypes by targeting the *fabB*, *fabA*, and *desA* genes and assessed their growth under a variety of conditions. As expected, these mutants displayed severely impaired growth profiles under both high- and low-pressure (HP and LP, respectively) conditions as well as an auxotrophic requirement for exogenous unsaturated fatty acids (UFA). Unexpectedly, extended incubation of these mutants led to the appearance of suppressor strains which no longer required exogenous UFA for growth and had growth phenotypes similar to that of wild-type *P. profundum* SS9 concomitant with greatly increased PUFA, up to 8-fold higher than for the wild type. We isolated and characterized these suppressor strains and subsequently resequenced their genomes to determine the genetic basis for these suppressor phenotypes. Of the four suppressor strains characterized, three contained mutations resulting in either impaired transcription or loss of function of *fabD*, which encodes the malonyl coenzyme A (malonyl-CoA)-ACP transacylase (MAT) of the type II FAS. Further experiments demonstrated that *fabD* is not an essential gene in *P. profundum* SS9 and that the *ΔfabD* mutation is synthetically lethal with mutations in *pfaA*, which contains a homologous FabD domain. We further demonstrated that loss of *fabD* in E. coli can be complemented by heterologous expression of Pfa synthases from *P. profundum* SS9 and Shewanella pealeana.

## RESULTS

### Fatty acid biosynthesis genes in *P. profundum* SS9.

Analysis of the Photobacterium profundum SS9 genome identified the genes associated with the prototypical type II FAS similar to that found in E. coli. A full list of predicted fatty acid biosynthesis genes is given in Table S1. *P. profundum* SS9 contains both *fabA* and *fabB* homologs involved in the biosynthesis of monounsaturated fatty acids. In addition to the canonical type II FAS genes, *P. profundum* SS9 also contains a type I FAS/polyketide synthase that is responsible for the biosynthesis of the polyunsaturated fatty acid eicosapentaenoic acid (EPA; 20:5*n-*3). The genome also contains a homolog of *desA*, encoding a membrane-bound oxygen-dependent desaturase, which has been shown to be involved in the production of monounsaturated fatty acids in other *Gammaproteobacteria* (22–24). Another interesting feature is a cluster of putative fatty acid biosynthesis genes present on chromosome 2 that bears a striking resemblance to the O138 genomic island present in pathogenic strains of E. coli (25, 26). It is unknown whether these genes contribute to fatty acid biosynthesis or if they encode catalytically active proteins.

### Phenotypes associated with loss of MUFA-related genes.

**FIG 2:**
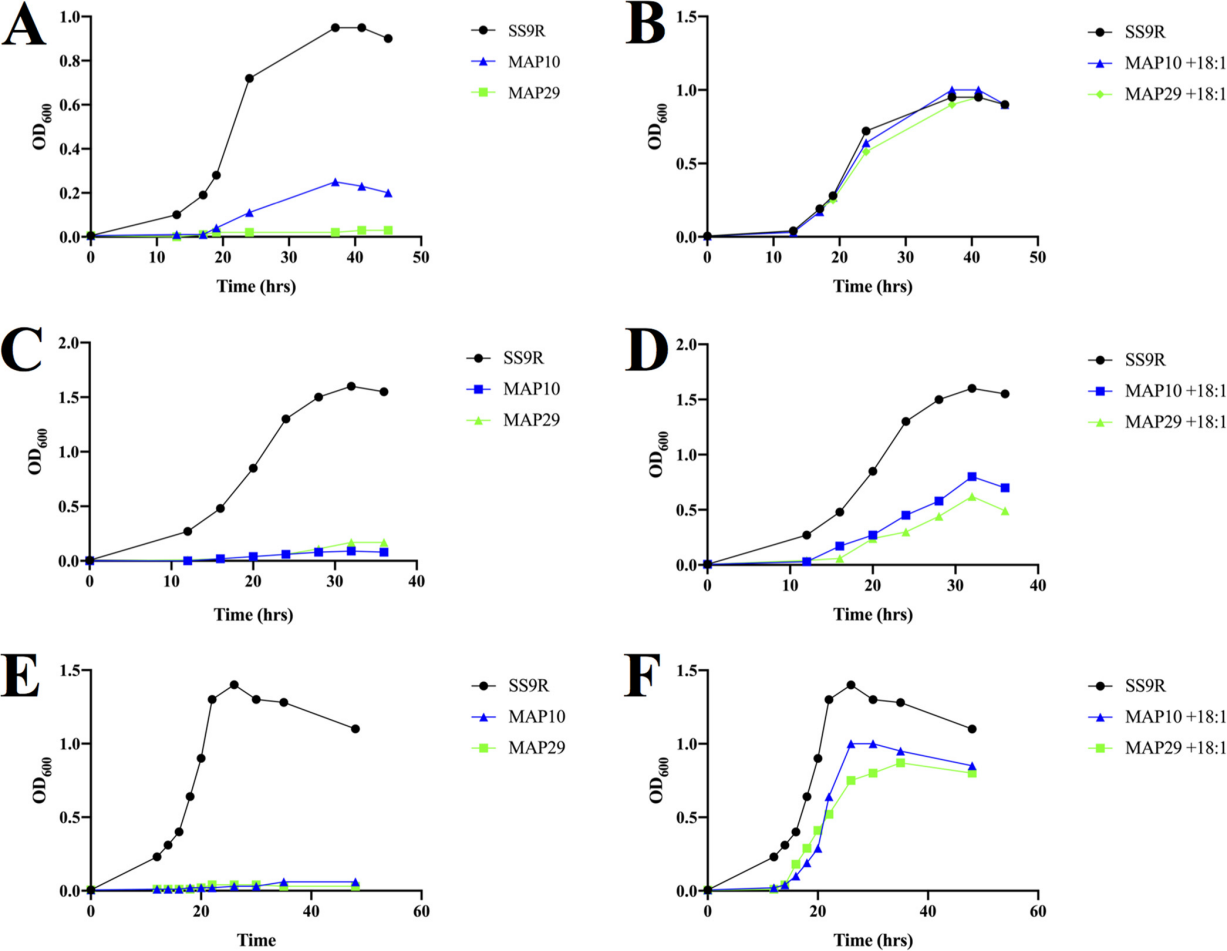
Growth phenotypes associated in MUFA gene deletion mutants. Growth of MAP10 (*ΔfabB*) and MAP29 (*ΔfabA ΔdesA*) strains is severely impaired at 15°C (A), 30 MPa (C), and 0.1 MPa (E). Growth phenotypes of mutant strains can be complemented by the addition of 0.05% Tween 80 (18:1) to growth media at 15°C (B), 30 MPa (D), and 0.1 MPa (F). All pressure experiments were performed at 15°C.

Given the presence of the Pfa synthase, we predicted that genes related to MUFA biosynthesis in SS9 would be nonessential. To test this hypothesis, we generated in-frame deletion mutants MAP10 (*ΔfabB*) and MAP29 (*ΔfabA ΔdesA*) (Table 1) that had various loss-of-function phenotypes in MUFA biosynthesis. The growth phenotypes of these strains under various culture conditions are shown in Fig. 2. The MAP10 (*ΔfabB*) strain was capable of limited growth under aerobic conditions (Fig. 2A) but displayed virtually no growth in the microaerobic sealed bulbs used in pressure experiments (Fig. 2C and E). To obtain a strain completely dependent on exogenous monounsaturated fatty acids, strain MAP29 (*ΔfabA ΔdesA*) was constructed. Similar to previous work in Pseudomonas aeruginosa (22), deletion of both *fabA* and *desA* led to an obligate requirement for exogenous MUFA supplementation. Supplementing the growth medium with 18:1 fatty acid in the form of Tween 80, which has been shown to be an effective means of delivering fatty acids to SS9 (7, 27), fully or partially complemented the growth phenotypes of both mutants as expected (Fig. 2B, D, and F.) The limited growth of MAP10 (*ΔfabB*) allowed for fatty acid analysis of the strain grown at 15°C (Table 2). This strain displayed the expected phenotype of severely limited amounts of MUFA and an approximate 4-fold increase in EPA (20:5*n-*3) content relative to that of wild-type SS9R. This strain also exhibited increased proportions of myristic (14:0) and palmitic (16:0) fatty acids.

**TABLE 1:** Bacterial strains and plasmids used in this study

Strain	Relevant characteristics	Reference or source
Escherichia coli strains
DH5α*pir*	*recA* mutant; cloning strain used for R6K plasmids	56
MG1655	Wild type	CGSC^*a*^
DY329	W3110 Δ*lacU169 nadA*::Tn*10 gal490* λcI857(*cro-bioA*)	35
BW25113	(*araD-araB*)567 *lacZ*4787D::*rrnB*-4 *lacI*^q^ *rpoS*396 *rph*-1 D(*rhaD-rhaB*)568 *rrnB*-4 *hsdR514*	57
LA2-89	*fabD89* (ts)	CGSC
MAE20	MG1655 *ΔfabB*::Kan^r^	This study
MAE21	BW25113 1F12R	58
MAE30	MG1655 *ΔfabA*::Cm^r^	This study
MAE41	BW25113 pFOS8E1	This study
MAE46	DY329 *ΔfabD*::Kan^r^ pMA84	This study
MAE47	BW25113 *ΔfabD*::Kan^r^ 1F12R	This study
MAE48	BW25113 *ΔfabD*::Kan^r^ pFOS8E1	This study
Photobacterium profundum strains
SS9R	Rifampicin-resistant derivative of *P. profundum* SS9	49
EA2	Chemically derived MUFA auxotroph	7
MAP10	SS9R *ΔfabB*	This study
MAP16	SS9R *ΔlacZ ΔpfaA*::*lacZY*	27
MAP28	SS9R *ΔdesA*	This study
MAP29	MAP28 *ΔfabA*	This study
MAP37	SS9R Δ*fabD*	This study
MAP41	MAP29 *fabD* disruption mutant	This study
MAP1002	MAP10 high-pressure-derived suppressor	This study
MAP1003	MAP10 high-pressure-derived suppressor	This study
MAP2902	MAP29 high-pressure-derived suppressor	This study
MAP2903	MAP29 high-pressure-derived suppressor	This study
Plasmids		
pRE118	Suicide allelic-exchange plasmid, R6K origin; Kan^r^ *sacB*	59
pMUT100	Suicide plasmid insertional inactivation, Kan^r^	60
pKT231	Broad-host-range plasmid; Kan^r^ Sm^r^	61
pFL122	Broad-host-range plasmid; Sm^r^	62
pBAD24	Expression vector; arabinose inducible	63
pKD46	Arabinose inducible λ Red functions; Amp^r^	52
pKD3	Source of Cm^r^ cassette for recombineering	25
pKD4	Source of Kan^r^ cassette	25
pCC2FOS	Fosmid cloning vector	Epicentre
1F12R	pCC2FOS w/*pfaABCDE* from Shewanella pealeana	58
pFOS8E1	Fosmid clone w/*pfaABCD* from *P. profundum* SS9	36
pEA44	pKT231 containing SS9R *fabF*	9
pEA30	pMUT100 containing *pfaA* internal fragment	7
pEA101	pMUT100 containing *pfaD* internal fragment	36
pMA31	pRE118 containing SS9R *ΔfabB* allele	This study
pMA55	pFL122 containing SS9R *fabB*	This study
pMA56	pFL122 containing SS9R *fabA^M77T^*	This study
pMA57	pFL122 containing SS9R *fabA^WT^*	This study
pMA58	pRE118 containing SS9R *ΔfabA* allele	This study
pMA64	pRE118 containing SS9R *ΔdesA* allele	This study
pMA71	pMUT100 containing SS9R *fabD* internal fragment	This study
pMA79	pRE118 containing SS9R *ΔfabD* allele	This study
pMA84	pBAD24 with E. coli *fabD* under pBAD control	This study

a CGSC, Coli Genetic Stock Center, Yale University.

**TABLE 2:** Fatty acid profiles of SS9R, MAP10 (*ΔfabB*), and MAP37 (Δ*fabD*) at 15°C

Fatty acid	Mean % fatty acid^*a*^		
	SS9R	MAP10 (*ΔfabB*)	MAP37 (*ΔfabD*)
12:0	4.06 ± 1.47	8.19 ± 0.14	3.07 ± 0.19
14:0	4.17 ± 1.09	22.32 ± 0.45	5.49 ± 0.50
14:1	3.24 ± 1.07	0.58 ± 0.50	1.81 ± 0.16
16:0iso	3.33 ± 0.96	0.88 ± 0.03	2.16 ± 0.76
16:0	23.04 ± 3.34	36.01 ± 0.82	27.76 ± 0.67
16:1	43.29 ± 2.51	3.39 ± 0.12	37.18 ± 2.52
12-OH	1.79 ± 1.14	3.01 ± 0.02	2.02 ± 0.83
18:0	0.63 ± 0.20	0.26 ± 0.22	1.25 ± 0.26
18:1	11.47 ± 3.23	0.00 ± 0.00	8.09 ± 0.73
20:5	4.98 ± 1.18	20.84 ± 1.67	11.18 ± 2.59
22:6	0.00 ± 0.00	0.00 ± 0.00	0.00 ± 0.00

a Data represent the averages ± standard deviations of triplicate samples.

### Suppressor mutations in *fabA* and *fabB* mutant strains.

**FIG 3:**
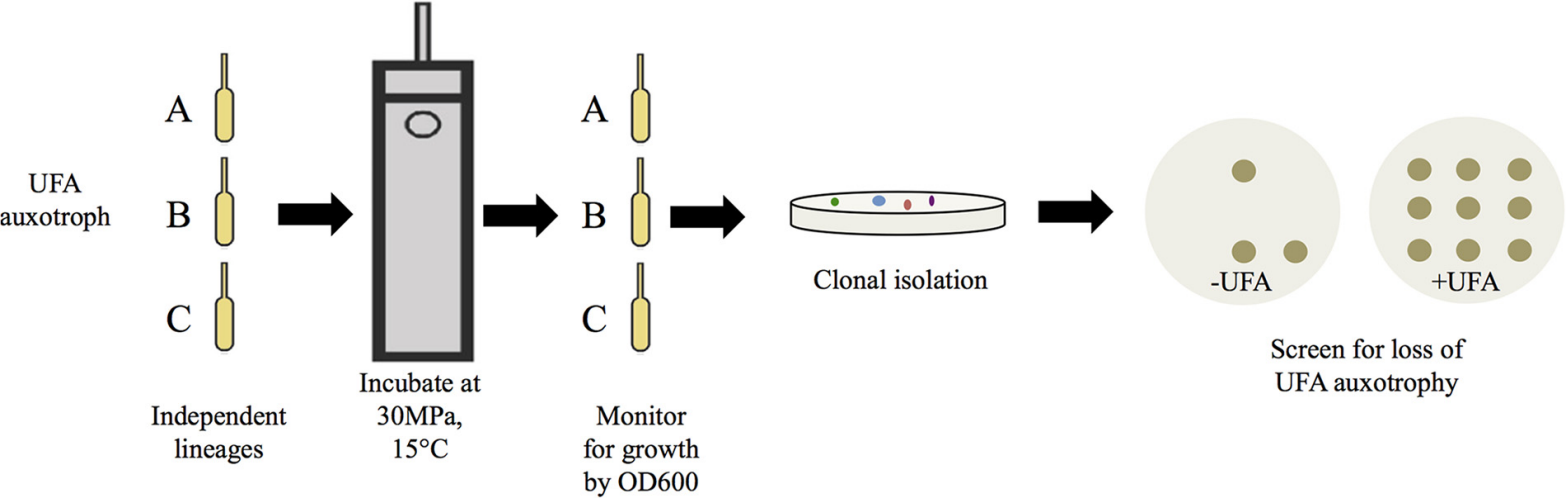
Schematic diagram of the selection and isolation of high-pressure-derived suppressor strains in this study. Multiple independent lineages (A, B, and C) of strains MAP10 (*ΔfabB*) and MAP29 (*ΔfabA ΔdesA*) were incubated in heat-sealed pressure bulbs at 30 MPa. Periodic samplings were performed to check for growth by OD_600_. Significant increases in growth ascertained by optical density readings were indicative of the appearance of suppressor strains, and bulbs with growth were selected for clonal isolation on solid medium. Isolated colonies were screened for loss of UFA auxotrophy on 2216 marine agar with or without 0.05% Tween 80 (18:1).

During the characterization of these mutants, we observed that extended incubation (>2 weeks) of these cultures under a variety of conditions led to prominent growth similar to that of the parental strain SS9R. Serial dilution and plating of these cultures onto solid media indicated the presence of small and large colonies. Upon isolation of both colony types, it was noted that the smaller colonies retained an UFA auxotrophy phenotype, while the larger colonies grew equally well on 18:1-supplemented and nonsupplemented media. This phenomenon indicated the possibility of second site suppressor mutations that allow for growth in the absence of MUFA biosynthesis. Given our interest in the interplay between MUFA and PUFA biosynthesis and how both pathways are affected by parameters such as hydrostatic pressure, we devised a genetic selection scheme, outlined in Fig. 3, to isolate suppressor mutants from MAP10 (*ΔfabB*) and MAP29 (*ΔfabA ΔdesA*) genetic backgrounds at elevated hydrostatic pressure. This condition was chosen for isolation of suppressor mutants due to the complete lack of growth of these mutants at 30 MPa. Furthermore, previous work in *Photobacterium profundum* SS9 had demonstrated that high-pressure growth was distinctly affected by disruption in MUFA biosynthesis (7). Following this scheme, we isolated two independent suppressor strains from each genetic background and subjected them to detailed analyses as described below.

**FIG 4:**
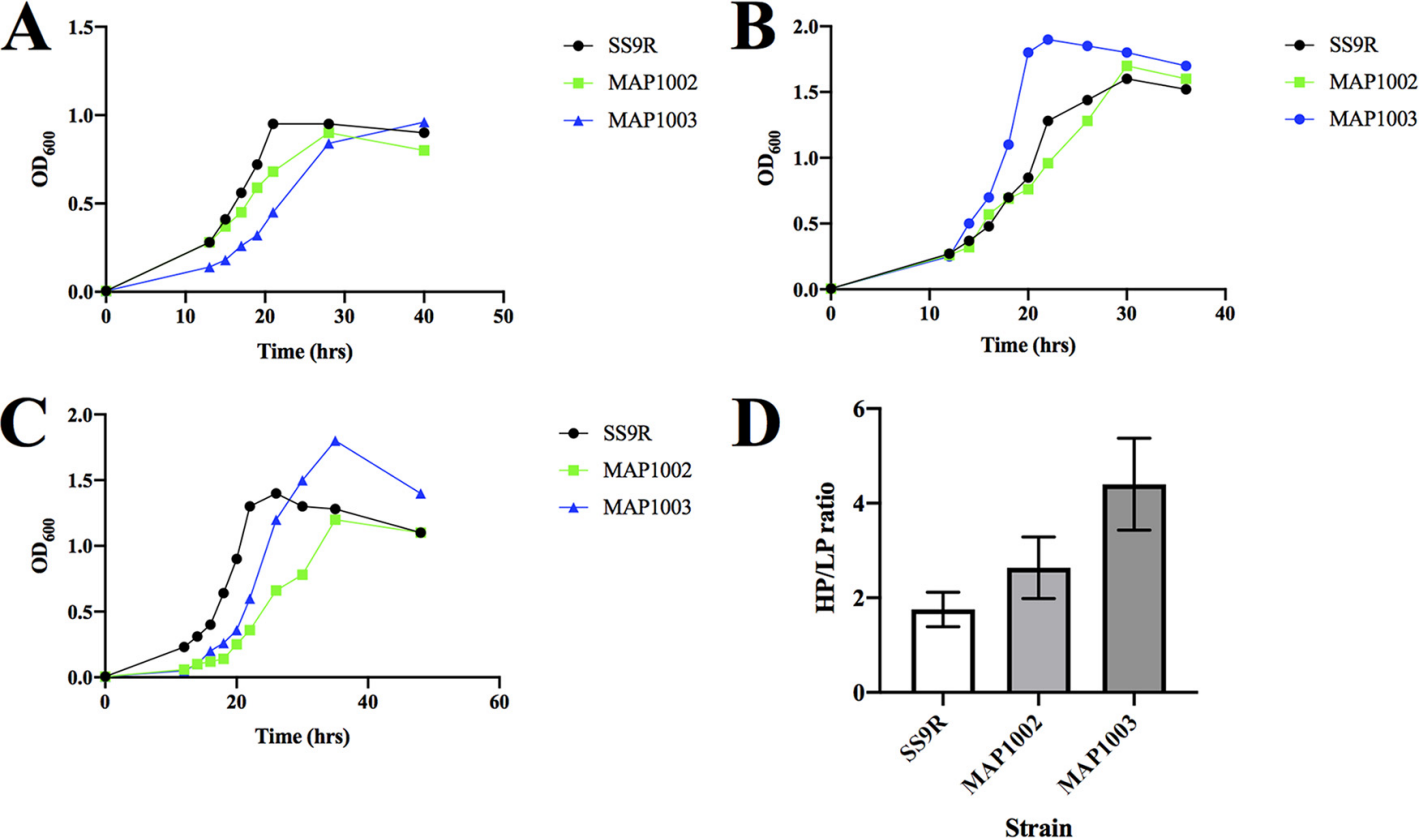
Growth analyses of MAP10 (*ΔfabB*)-derived suppressor strains under various culture conditions. (A to C) Growth curves of SS9R, MAP1002, and MAP1003 at 15°C (A), 30 MPa (B), and 0.1 MPa (C). (D) High-pressure/low-pressure (HP/LP) ratios of SS9R, MAP1002, and MAP1003 grown at 15°C. Results are the means of at least three independent experiments, with error bars signifying 1 standard deviation.

Growth analyses for the MAP10 (Δ*fabB*)-derived suppressor strains at high and low pressure and in standard aerobic tubes are presented in Fig. 4. Results indicate that both MAP1002 and MAP1003 are capable of growth under conditions that did not support growth of the parental strain MAP10 (*ΔfabB*). Comparison of these suppressor growth profiles also indicated that these profiles are similar to that of wild-type SS9R under the various conditions tested. Analysis of piezophily, expressed as the HP/LP ratio, is the result of dividing optical densities of identical cultures grown at both 0.1 MPa and 30 MPa. By this metric, values >1 represent piezophilic growth and those <1 represent piezosensitivity. As expected from previous work in this strain (28), the HP/LP ratio of SS9R was ∼1.8, indicative of piezophilic growth. A comparison of the wild type and both suppressor strains derived from MAP10 (*ΔfabB*) is shown in Fig. 4D and indicated that relative to SS9R, both MAP1002 and MAP1003 displayed significantly enhanced high-pressure growth. Fatty acid profiles of MAP1002 and MAP1003 grown under various conditions are presented in Table 3. Comparison of these profiles to that of MAP10 (*ΔfabB*), the parental strain, indicated that both suppressor strains had recovered the ability to produced MUFA to various degrees. Both strains also maintained elevated proportions of the omega-3 fatty acid EPA (20:5 *n-*3) relative to that of SS9R as well as the appearance of the omega-3 PUFA docosahexaenoic acid (DHA; 22:6 *n-*3) in MAP1003.

**TABLE 3:** Fatty acid profiles of MAP10 (*ΔfabB*)-derived suppressors under indicated conditions

Fatty acid	Mean % fatty acid^*a*^					
	MAP1002			MAP1003		
	15°C	0.1 MPa^*b*^	30 MPa^*b*^	15°C	0.1 MPa^*b*^	30 MPa^*b*^
12:0	7.68 ± 1.10	6.60 ± 0.83	4.21 ± 0.79	7.38 ± 0.68	6.48 ± 0.19	4.05 ± 0.14
14:0	14.32 ± 2.84	20.50 ± 1.27	9.35 ± 1.18	20.46 ± 3.32	16.81 ± 0.71	5.55 ± 0.98
14:1	1.69 ± 0.51	1.31 ± 0.51	1.18 ± 0.15	0.00 ± 0.00	0.00 ± 0.00	0.00 ± 0.00
16:0iso	1.67 ± 0.62	0.26 ± 0.45	0.60 ± 0.53	1.00 ± 0.31	0.78 ± 0.68	0.55 ± 0.49
16:0	30.22 ± 3.36	50.12 ± 6.10	47.37 ± 4.54	33.54 ± 1.36	44.66 ± 0.85	40.82 ± 1.06
16:1	24.82 ± 3.03	8.05 ± 0.78	11.58 ± 1.32	15.40 ± 1.53	2.04 ± 0.65	3.71 ± 1.52
12-OH	2.93 ± 0.73	2.37 ± 0.91	2.56 ± 1.31	4.17 ± 0.85	3.66 ± 0.27	3.45 ± 0.08
18:0	0.98 ± 0.01	0.37 ± 0.32	1.25 ± 0.25	0.63 ± 0.08	0.50 ± 0.10	1.58 ± 1.46
18:1	1.45 ± 0.16	0.53 ± 0.45	2.15 ± 0.27	0.33 ± 0.29	0.00 ± 0.00	0.71 ± 0.60
20:5	14.25 ± 3.03	9.91 ± 3.35	19.76 ± 0.65	17.11 ± 0.32	24.95 ± 1.76	37.34 ± 0.87
22:6	0.00 ± 0.00	0.00 ± 0.00	0.00 ± 0.00	0.00 ± 0.00	0.11 ± 0.20	2.23 ± 0.16

a Data represent the averages ± standard deviations of triplicate samples.

b Pressure incubations were performed in heat-sealed bulbs at 15°C.

**FIG 5:**
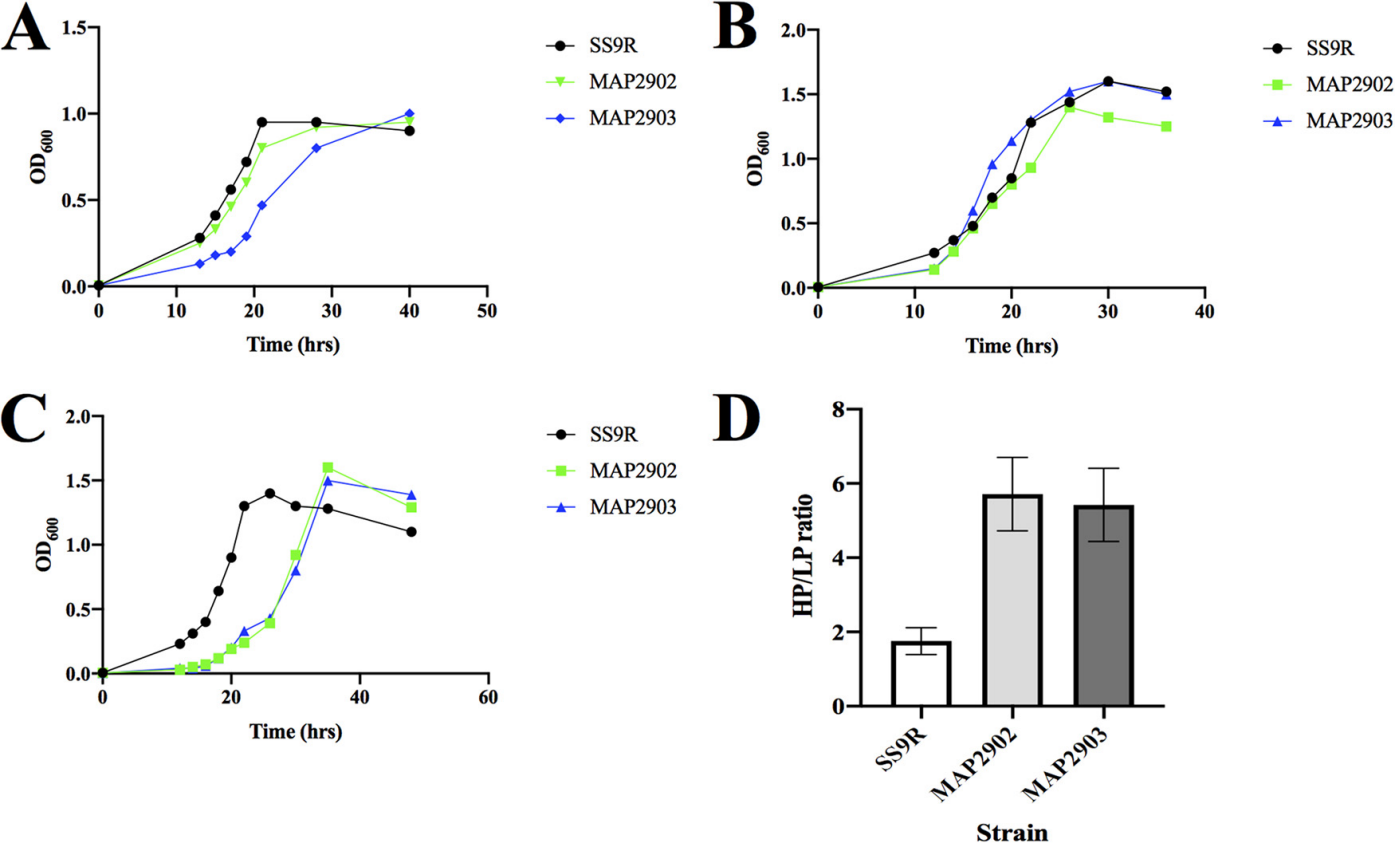
Growth analyses of MAP29 (*ΔfabA ΔdesA*)-derived suppressor strains under various culture conditions. (A to C) Growth curves of SS9R, MAP2902, and MAP2903 at 15°C (A), 30 MPa (B), and 0.1 MPa (C). (D) HP/LP ratios of SS9R, MAP2902, and MAP2903 grown at 15°C. Results are the means of at least three independent experiments, with error bars signifying 1 standard deviation.

Growth (Fig. 5) and fatty acid (Table 4) profiles were also obtained for suppressor strains MAP2902 and MAP2903, which share the same parental strain, MAP29 (*ΔfabA ΔdesA*). Similar to the trend seen with the MAP10 (*ΔfabB*)-derived suppressors, both MAP2902 and MAP2903 were capable of growth similar to that of wild-type SS9R at 15°C (Fig. 5A) and 30 MPa (Fig. 5B). Interestingly, both suppressor strains displayed impaired growth compared to that of SS9R at 0.1 MPa (Fig. 5C). The elevated HP/LP ratios observed for both MAP2902 and MAP2903 relative to that of SS9R (Fig. 5D) indicate that these strains have become relatively more piezophilic than their original parental strain, SS9R. Fatty acid profiles of these suppressor strains are presented in Table 4. The fatty acid profile of MAP29 (*ΔfabA ΔdesA*) was unable to be accurately obtained due to the obligate requirement for exogenous fatty acid supplementation for growth (Fig. 2A). Both suppressor strains contained significantly larger amounts of EPA (>30% of total fatty acid) as well as significant amounts of DHA, up to >3% of total cellular fatty acids, under all culture conditions. Unlike the MAP10 (*ΔfabB*)-derived suppressors, both MAP2902 and MAP2903 contained no MUFA, with the unsaturated fatty acid content being derived completely from EPA and DHA (up to >43% of total fatty acids).

**TABLE 4:** Fatty acid compositions of MAP29 (*ΔfabA ΔdesA*) suppressors under indicated conditions

Fatty acid	Mean % fatty acid^*a*^					
	MAP2902			MAP2903		
	15°C	0.1 MPa^*b*^	30 MPa^*b*^	15°C	0.1 MPa^*b*^	30 MPa^*b*^
12:0	4.46 ± 0.14	3.52 ± 0.13	3.69 ± 0.11	4.09 ± 0.18	3.61 ± 0.22	3.90 ± 0.07
14:0	12.86 ± 1.26	19.38 ± 1.15	9.31 ± 0.57	11.91 ± 0.88	18.17 ± 0.83	9.29 ± 0.10
14:1	0.00 ± 0.00	0.00 ± 0.00	0.00 ± 0.00	0.00 ± 0.00	0.00 ± 0.00	0.00 ± 0.00
16:0iso	1.10 ± 0.16	0.42 ± 0.20	0.61 ± 0.24	1.06 ± 0.12	0.00 ± 0.00	0.95 ± 0.19
16:0	37.44 ± 2.14	41.85 ± 0.43	41.32 ± 1.60	37.40 ± 2.47	40.12 ± 0.40	39.07 ± 0.43
16:1	0.00 ± 0.00	0.00 ± 0.00	0.00 ± 0.00	0.00 ± 0.00	0.00 ± 0.00	0.00 ± 0.00
12-OH	3.39 ± 0.69	1.88 ± 0.18	2.21 ± 0.61	3.31 ± 0.61	1.02 ± 0.40	3.14 ± 0.41
18:0	2.95 ± 0.91	0.92 ± 0.02	0.67 ± 0.03	3.12 ± 0.53	0.88 ± 0.05	0.62 ± 0.02
18:1	0.00 ± 0.00	0.00 ± 0.00	0.00 ± 0.00	0.00 ± 0.00	0.00 ± 0.00	0.00 ± 0.00
20:5	35.29 ± 1.77	30.82 ± 1.50	40.66 ± 2.48	35.96 ± 1.66	35.18 ± 0.47	41.86 ± 0.10
22:6	2.52 ± 0.74	0.65 ± 0.05	1.28 ± 0.29	3.16 ± 0.89	1.02 ± 0.11	1.17 ± 0.12

a Data represent the averages ± standard deviations of triplicate samples.

b Pressure incubations were performed in heat-sealed bulbs at 15°C.

### Identification of suppressor mutations.

Based on the phenotypic data collected, we performed whole-genome resequencing on these suppressor mutant strains to identify causal mutations. After barcode trimming and quality filtering, Illumina reads were mapped to the SS9 genome using the *breseq* pipeline (29). Genome coverage depth ranged from ∼70- to ∼250-fold, with >99% of the filtered reads mapping to the *P. profundum* genome. A complete list of mutations identified in these strains is provided in File S1. As expected, the *breseq* pipeline identified the *fabB*, *fabA*, and *desA* deletion mutations in the respective suppressor strains.

**FIG 6:**
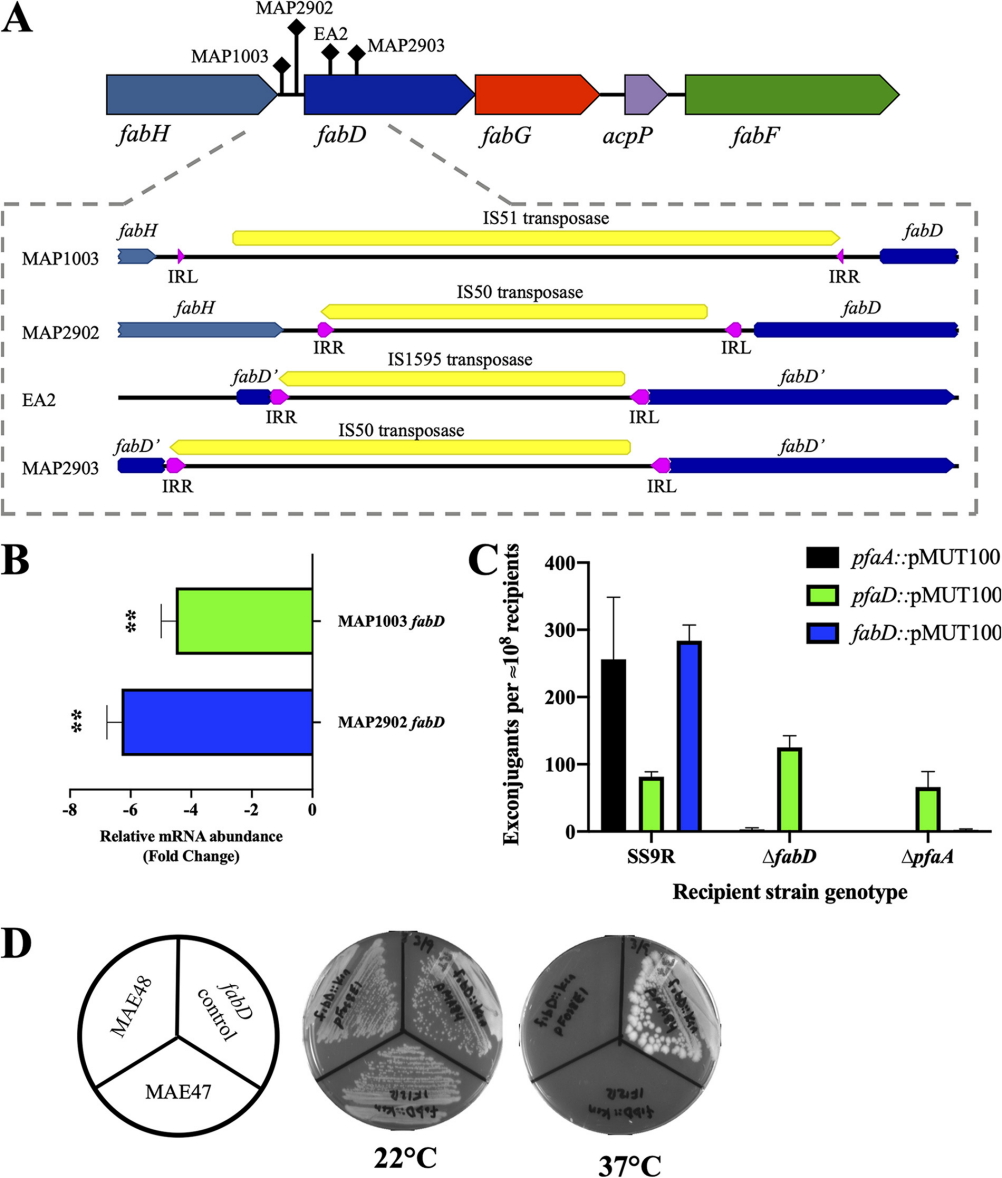
Various mutations in suppressor strains map to the *fabD* locus. (A) Diagram of the *fabHDG* operon of *P. profundum* SS9. Arrows indicate the locations of insertion sequences found in various suppressor mutants and the EA2 strain. The expansion shows detailed maps of the loci found in the indicated suppressor strain. Left (IRL) and right (IRR) inverted repeats are shown in pink, and various transposase coding sequences are shown in yellow. (B) Abundances of *fabD* mRNA in the indicated suppressor mutants relative to SS9R, as determined by qRT-PCR. (C) *ΔfabD* and *ΔpfaA* mutations are synthetically lethal in *P. profundum* SS9. Conjugation was used to mobilize insertional inactivation vectors into the indicated recipient strains. Values (±standard deviations) represent the average numbers of exconjugants obtained from three independent experiments. (D) Heterologous expression of the Pfa synthase at 22°C and not 37°C from either Shewanella pealeana (MAE47) or *P. profundum* SS9 (MAE48) complements the *fabD*::Kan^r^ mutation in E. coli. Strain MAE46 lacks the chromosomal copy of *fabD* and is complemented by *fabD* cloned on construct pMA84. Plates were imaged after 48 h at either 22°C (left) or 37°C (right).

Among the mutations identified in the suppressor strains, we noted insertion sequences (IS) that had integrated upstream or within the coding sequence of the *fabD* gene, which encodes the malonyl-CoA-ACP transacylase (MAT) enzyme involved in fatty acid biosynthesis. The diagram shown in Fig. 6A indicates the relative positions of these IS within the *fabHDG* operon. Reanalysis of EA2, a previously isolated chemical mutant of SS9 with reduced monounsaturated fatty acid and elevated EPA content (7), revealed a similar IS element integration into the *fabD* coding sequence. As shown in the bottom portion of Fig. 6A, each IS element contained one open reading frame encoding a predicted transposase/integrase flanked by inverted repeat sequences ranging from 16 to 50 bp. Homology searches of the IS elements found in MAP2902 and MAP2903 against a dedicated bacterial IS element database (30) indicated that they fall within the IS*4* family and are members of the IS*50* group. The genome of *P. profundum* SS9 contains 48 copies (>95% amino acid identity) of this IS*50* transposase, with 20 copies residing on the major chromosome (chromosome 1) and the additional 28 copies found on the minor chromosome (chromosome 2). The IS element in EA2 was homologous to the IS*1595* family, and a total of 9 copies (>99% nucleotide identity) were found in the SS9 genome (5 in chromosome 1 and 4 in chromosome 2). The IS element found in MAP1003 appears to be a member of the IS*3* family and IS*51* group and 14 copies (>95% nucleotide identity) were identified in the SS9 genome, with 7 on each of the chromosomes. The finding of mutations due to IS dynamics localized to the same locus in suppressor strains derived from different genetic backgrounds and in a chemical mutant with growth and fatty acid phenotypes similar to those of the suppressors indicates that mutation of *fabD* has a dominant impact in alleviating genetic defects in MUFA biosynthesis and not specific to a particular step in the type II FAS.

### FabD is not essential in SS9R.

The *fabD* gene is recognized as essential in bacteria due its role in producing malonyl-ACP, the extender unit used by ketosynthase enzymes for successive rounds of acyl chain elongation during fatty acid biosynthesis (13, 31). To confirm that *fabD* is indeed a nonessential gene in SS9R, we attempted to generate an in-frame deletion mutant of *fabD* in SS9R. This deletion was designed to remove 441 bp of the *fabD* coding sequence (∼48%) and included all of the known catalytic residues. To our surprise, the mutant was readily generated; it was designated MAP37 (Δ*fabD*) and displayed growth patterns identical to those of SS9R under a variety of culture conditions (data not shown). Fatty acid analyses of MAP37 (Δ*fabD*) showed that besides the ∼2-fold increase in EPA content, its fatty acid profile was remarkably similar to that of SS9R (Table 2). Given the locations of the various IS elements directly upstream of *fabD* in two of the suppressor strains, it is possible that these mutations might alter transcription of *fabD.* Quantification of *fabD* transcripts in both MAP1003 (*ΔfabB* suppressor) and MAP2902 (*ΔfabA ΔdesA* suppressor) indicated that both strains had severalfold decreases in *fabD* transcript abundance relative to that of SS9R (Fig. 6B).

To ascertain whether these mutations in *fabD* are indeed part of the genetic suppression mechanism, a null mutation in *fabD* was introduced into the parental MUFA auxotroph MAP29 (*ΔfabA ΔdesA*) via insertional inactivation. Analyses of the growth and fatty acid profiles of the resulting strain (MAP41) indicated that the strains were phenotypically identical to the suppressor strains MAP2902 and MAP2903 (Table S3).

### PfaA MAT domain can replace FabD function *in vivo*.

A comprehensive search of the *P. profundum* SS9 genome revealed that the MAT domain in PfaA is the only other protein within SS9 that contains predicted MAT activity (32, 33). Alignments of various FabD homologs and MAT domains from various Pfa synthases indicated that the catalytic residues associated with MAT activity are conserved among these domains/proteins (Fig. S2). Therefore, it was hypothesized that the MAT domain within PfaA can replace FabD function *in vivo*. To investigate this, we attempted to construct a mutant of *P. profundum* SS9 deficient in both *pfaA* and *fabD*. In these experiments, an in-frame deletion of one gene was paired with insertional inactivation of the other gene. Despite numerous attempts to recover exconjugants in a variety of genetic backgrounds, we were unable to recover exconjugants that contained mutations in both *fabD* and *pfaA* (Fig. 6C). Conversely, we were able to routinely recover exconjugants in genetic crosses targeting *pfaD*, the distal gene of the *pfa* operon containing a single enzymatic domain encoding enoyl reductase activity, utilizing recipient strains with *ΔfabD* and *ΔpfaA* genotypes. Given these results, we concluded that *fabD* and *pfaA* form a synthetic lethal pair in *P. profundum* SS9.

To further verify this interpathway complementation, we attempted to complement the well-characterized *fabD* mutation in E. coli LA2-89, which contains a temperature-sensitive *fabD89* allele. During our initial experiments with this strain, we noted the appearance of temperature-resistant suppressor mutants during routine strain passaging. Isolation of several non-temperature-sensitive mutants and subsequent PCR amplification and sequencing of the chromosomal *fabD* copy in these strains indicated that they all retained the *fabD89* allele characterized previously (34). These results indicate that loss of FabD activity in E. coli can be suppressed via a yet-to-be-characterized mechanism.

To prevent these suppressor mutations from obscuring these results, the chromosomal copy of *fabD* in E. coli was replaced with a kanamycin resistance cassette in the presence of a cloned copy of *fabD* residing on a plasmid (pMA84) using λ Red gene replacement (35). P1 phage lysates were derived from the resulting strain (MAE46) and used in transductions with various recipient strains carrying cloned Pfa synthases. Recipient cells containing the *fabD* expression plasmid yielded transductant colonies as expected. Additionally, the *fabD*::Kan^r^ mutation was also able to be successfully transduced into recipient cells carrying fosmids with cloned Pfa synthases originating from either *P. profundum* SS9 (pFOS8E1) or Shewanella pealeana (1F12R) (Fig. 6D). While complementation of the *fabD*::Kan^r^ mutation with the *fabD* construct (pMA84) occurred under all temperatures as expected, complementation supplied by either Pfa synthase was temperature sensitive, with no growth observed at 37°C (Fig. 6D). This complementation also occurs in the absence of EPA synthesis, as the pFOS8E1 construct lacks the specific phosphopantetheinyl transferase (*pfaE*) required for EPA production under all conditions (36).

### Other suppressor mutations of interest.

Of the suppressor strains characterized, MAP1002 (*ΔfabB* suppressor) caught our attention due to its unique fatty acid profile, which contained increased amounts of palmitoleic acid (16:1) despite the absence of FabB. Resequencing of this mutant revealed a nonsynonymous mutation (M77T) in *fabA*, encoding the dual-function dehydratase/isomerase that is primarily responsible for anaerobic generation of MUFA in most Gram-negative bacteria (22, 37, 38). Based on sequence alignments, this methionine residue is highly conserved among FabA homologs and is in close proximity to the FabA active-site residue. As shown in Fig. S3A in the supplemental material, both *fabA^M77T^* and *fabA^WT^* were cloned onto pFL122 and both complemented the *ΔfabA* mutation in E. coli strain MAE30 (*ΔfabA*::Cm^r^) equally well. Fatty acid analysis of these strains indicated that the complemented strain carrying the *fabA^M77T^* variant produced more monounsaturated fatty acids than the *fabA^WT^* variant, particularly at lower temperatures (Fig. S3B). Given that both *fabA* variants were cloned identically and carried in the same strain, it appears that the M77T mutation alters the activity of FabA in some yet-to-be-characterized way.

Given the increase in MUFA content in MAP1002 relative to that in MAP10, another ketosynthase enzyme might substitute for FabB activity in *P. profundum* SS9. A similar study performed in Shewanella oneidensis MR-1 indicated that suppression of *ΔfabB* occurred by mutations that increased transcription of *fabF* (23). To determine if *fabF* from SS9R could indeed replace FabB activity, we generated a *ΔfabB*::Kan^r^ mutant of E. coli (MAE20) and introduced pMA55 and pEA44 (9), which contain cloned copies of *fabB* and *fabF*, respectively, from SS9. Based on growth on solid medium, both FabB and FabF from *P. profundum* SS9 could complement the *ΔfabB*::Kan^r^ mutation in E. coli (see Fig. S3C in the supplemental material).

## DISCUSSION

In this study, we utilized a genetic approach to gain insights into the metabolic interactions between the two fatty acid biosynthesis pathways in the PUFA-producing deep-sea bacterium Photobacterium profundum SS9. Based on its genome sequence, *P. profundum* SS9 contains the full complement of type II FAS genes required for the biosynthesis of SFA and MUFA in addition to the Pfa synthase complex required for omega-3 PUFA synthesis (see Fig. S1 in the supplemental material). At the genome level, it is interesting that in some cases, multiple homologs of particular *fab* genes or genes with overlapping activities are present. Given the presence of the Pfa synthase and its role in producing EPA in this strain, we predicted that loss of MUFA biosynthesis via genetic disruption of either *fabA/desA* or *fabB* would not be lethal. While genetic disruption of either set of genes proved to be possible, the resulting strains displayed various degrees of auxotrophy for unsaturated fatty acids. As expected from previous work (7, 27), providing exogenous oleic acid in the form of Tween 80 (18:1) reversed this growth impairment.

Despite the similarity between *P. profundum* SS9 and E. coli in terms of fatty acid biosynthesis genes, there are differences in the phenotypes of comparable mutants. While the loss of *fabB* in E. coli leads to complete MUFA auxotrophy, its loss in *P. profundum* SS9 does not result in a lethal phenotype. The fatty acid profile of MAP10 (*ΔfabB*) is consistent with FabB acting as the ketosynthase responsible for the majority of MUFA biosynthesis in *P. profundum* SS9. The small amount of MUFA remaining in the fatty acid profile indicates that a bypass for FabB function exists in *P. profundum* SS9. One possibility is that FabF, a ketosynthase primarily responsible 18:1 synthesis (9), can functionally replace FabB. Our finding that FabF from *P. profundum* SS9 can support limited growth of an E. coli *fabB* mutant is consistent with this notion and has also been observed with FabF from Shewanella oneidensis MR-1 (23). Another possibility is that DesA, an oxygen-dependent desaturase, acts upon saturated acyl chains within the membrane to generate the MUFA content observed. The difference in growth of MAP10 (*ΔfabB*) observed under aerobic versus microaerobic conditions (Fig. 2A and E) is consistent with the possibility that DesA could be involved in this bypass mechanism. Previous work in P. aeruginosa PAO1 has indicated that both FabB and FabA function can be bypassed by DesA (22).

Extended incubation of the various MUFA auxotrophs at elevated hydrostatic pressure led to suppression of this unsaturated fatty acid auxotrophy and growth similar to that of wild-type SS9R under various culture conditions. Isolation and phenotypic characterization of these high-pressure-derived suppressor strains indicated that increased EPA production was a common trait among these strains. The sustained lack of MUFA biosynthesis in some of these suppressor strains further indicates that high levels of EPA (>15% total fatty acid) are required to compensate for loss of MUFA biosynthesis. Consistent with this finding, attempts to genetically disrupt the Pfa synthase failed in these suppressor strains. Interestingly, some of the suppressor strains analyzed in this work also produced docosahexaenoic acid (DHA; 22:6*n-*3). No mutations were observed in any of the genes encoding the Pfa synthase (*pfaABCDE*) in these suppressor strains, indicating that DHA biosynthesis is not linked to a genetic change in the currently known *pfa* biosynthetic pathway.

Considering that three of the four suppressor strains analyzed contained mutations mapping to the *fabD* locus, we suggest that FabD plays an important role in the “cross talk” between the Pfa synthase and type II FAS. Presumably, loss of FabD would render more malonyl-CoA available for processing by the Pfa synthase MAT domain, leading to the increased production of EPA observed in these strains. Comparison of the fatty acid compositions of MAP37 (*ΔfabD*) and SS9R indicates that EPA production can be doubled, with only mild perturbations of SFA and MUFA content, indicating that the availability of malonyl-CoA might be a factor in determining how much EPA can be produced by the Pfa synthase. Indeed, the synthetic engineering of microbial strains for elevated fatty acid or polyketide products that require malonyl-CoA as an extender unit can be optimized by increasing the malonyl-CoA pool via overexpression of the acetyl-CoA carboxylase (*acc*) genes responsible for malonyl-CoA synthesis (39–41).

Loss or reduction of FabD function may also impact downstream aspects of Pfa synthase activity and incorporation of end product (EPA) into phospholipid. Research on type II FAS inhibition using the enoyl-reductase (FabI) inhibitor triclosan in the Gram-positive bacterium Staphylococcus aureus indicated that mutations arose in *fabD* as part of a exogenous fatty acid bypass mechanism (42, 43). In contrast to our results and in line with previous findings for E. coli (44), mutations in *fabD* led to an obligate fatty acid auxotroph phenotype. The loss of FabD was proposed to allow for accumulation of holo-ACP, which allowed for more efficient transfer of exogenous fatty acids to holo-ACP and, ultimately, into phospholipid. Recent biochemical characterization of PfaB suggests that the embedded acyltransferase domain releases the final product of the Pfa synthase as a free fatty acid (45). A similar competitive mechanism in which holo-ACP is required for efficient PUFA incorporation could also explain the suppressor mutations found in this work.

The finding that *fabD* is dispensable in *P. profundum* SS9 demonstrates that this gene is not essential for growth and viability. Our genetic experiments indicate that the MAT domain found in PfaA can act as a bypass for the loss of *fabD*. Heterologous complementation of the *fabD*::Kan^r^ allele in E. coli using cloned Pfa synthases from both *P. profundum* SS9 and Shewanella pealeana supports this model. The temperature sensitivity of this complementation is interesting in light of previous observations that EPA production, whether in a native strain or heterologous E. coli, is similarly sensitive to temperature (1, 2, 6, 10, 46). Specifically, PUFA production in the heterologous host, E. coli, is typically enhanced at lower temperatures that more closely match the optimal growth temperatures of PUFA-producing deep-sea and psychrophilic strains. Based on our results, EPA production is not responsible for the observed complementation in E. coli and suggests that the MAT domain within PfaA is capable of transferring the malonyl group from malonyl-CoA to both the tandem acyl carrier protein (ACP) domains on PfaA and the soluble ACP of the type II FAS. During the course of our work, a report showed that the MAT domain of PfaA loads malonyl units onto the adjacent ACP domains within PfaA *in vitro* (3).

FabD may not be essential in other bacteria containing a Pfa synthase or a homologous secondary lipid synthase. A search of results from a high-throughput transposon screen (47) indicated that *fabD* may not be essential in Shewanella loihica PV-4, which contains a Pfa synthase (10, 15) and presumably produces EPA. Homologs of the Pfa synthase with uncharacterized products have been noted in numerous distinct lineages of bacteria, and the majority of these synthases contain an embedded MAT domain (15). Interestingly, these findings are conversely related to previous results in Streptomyces glaucescens, for which the relaxed specificity of FabD allowed for malonyl-CoA loading of both the type II FAS and a type II polyketide synthase (48).

Another possible route of suppression was revealed in the analysis of strain MAP1002, which contains the *fabA^M77T^* allele but no disruption of *fabD*. While the effect of this mutation was not extensively explored in this work, our results in the heterologous host E. coli showed that the FabA^M77T^ variant produces more MUFA than does FabA^WT^ *P. profundum* SS9 (Fig. S3B). One possible scenario is that the FabA^M77T^ allele has altered isomerase activity which, in a *fabB* mutant background, allows the remaining ketosynthase, FabF, to elongate the *cis*-decenoyl-ACP (10:1) substrate to produce MUFA. The finding that FabF of *P. profundum* SS9 can partially complement the loss of E. coli *fabB* is consistent with this idea. While these results cannot definitively rule out the activity of DesA, the increased amount of MUFAs in MAP1002 relative to MAP10 (*ΔfabB*), even in microaerobic bulbs, is suggestive of at least a partial increase in the anaerobic capacity to produce MUFA.

Taken together, the results presented here indicate that the Pfa synthase found in many marine *Gammaproteobacteria* can significantly influence total fatty acid biosynthesis in these strains. These results indicate that elevated levels of polyunsaturated fatty acids alone (>30% of total) can indeed support growth of *P. profundum* SS9 under a variety of growth conditions. The genetic investigation of these strains uncovered new metabolic interactions between the Pfa synthase and type II FAS, including the discovery of a novel approach to amplify bacterial omega-3 PUFA synthesis by disrupting type II FAS functions, specifically *fabB* and *fabD*. The harboring of two separate fatty acid biosynthetic pathways that can individually support growth may provide a level of genetic redundancy to optimize fitness when one pathway is disrupted either through mutation or, possibly, by antimicrobial compounds that target fatty acid biosynthesis in the complex microbial communities where these species exist in nature.

## MATERIALS AND METHODS

### Bacterial strains and growth conditions.

Escherichia coli strains were routinely grown at 37°C in Luria-Bertani (LB) medium unless stated otherwise. Photobacterium profundum SS9 strains were grown at 15°C in 2216 marine broth (Difco) at 75% strength (28 g/liter). For solid media, agar was included at 15 g/liter. The antibiotics kanamycin (50 μg/ml for E. coli and 200 μg/ml for *P. profundum*), chloramphenicol (15 μg/ml), ampicillin (100 μg/ml), and rifampicin (100 μg/ml) were used as required. Fatty acid auxotroph strains were grown in medium supplemented with 0.05% Tween 80 as a source of fatty acids. Liquid cultures were maintained in standard glass test tubes in a shaking incubator at 15°C. For pressure-related growth experiments, *P. profundum* SS9 strains were grown in heat-sealed bulbs and incubated in stainless steel pressure vessels as described previously (49). For growth in heat-sealed bulbs, marine broth was supplemented with 0.4% glucose and 100 mM HEPES (pH 7.5). High-pressure/low-pressure (HP/LP) ratios were obtained as described previously (21, 28). Briefly, cells from 2-day cultures grown at 15°C in aerobic tubes were inoculated into a volume of medium (∼20 ml) and transferred to heat-sealed bulbs. Individually sealed bulbs were placed in either 30-MPa or 0.1-MPa pressure vessels and incubated at 15°C. When the LP (0.1 MPa) culture bulb entered early logarithmic phase (optical density at 600 nm [OD_600_], 0.1 to 0.4), two values were recorded: the OD_600_ of the 30-MPa culture (HP) and the OD_600_ of the 0.1-MPa culture (LP). Under these conditions, the HP/LP ratio of the wild-type SS9R strain was 1.5 to 2.

### Mutagenesis in *P. profundum* SS9.

In-frame deletions were generated by allelic exchange using the suicide vector pRE118. Briefly, upstream and downstream regions (∼500 to 1,000 bp) of genes to be deleted were amplified by PCR with primer pairs (upstream region, 5F and 5R; downstream region, 3F and 3R) listed in Table S2. PCR products were assembled via 20-bp homologies using Gibson assembly (New England BioLabs) and cloned into restriction sites of pRE118. Verified constructs were introduced into *P. profundum* strains by biparental conjugation using S17-1λ*pir* following previously described protocols (1). Exconjugants were selected for on 2216 agar containing rifampicin and kanamycin at 15°C. To select for deletion mutants, exconjugants were grown in the absence of antibiotic selection and dilutions were plated onto 2216 agar containing 5% sucrose (*sacB* counterselection) and 0.05% Tween 80 (18:1), if needed. Colonies were screened for kanamycin sensitivity, sucrose resistance, and fatty acid auxotrophy. PCR and DNA sequencing using primers (*Δ*gene ver F and R) flanking the gene(s) of interest were used to confirm deletions. For insertional inactivation, internal fragments of genes to be disrupted were amplified by PCR and cloned into pMUT100 using standard molecular techniques (50). Derivatives of pMUT100 were mobilized into *P. profundum* SS9 by biparental conjugation using E. coli MC1061 containing pRK24 and pRL528 plasmids as described previously (51).

### Mutagenesis of Escherichia coli.

Gene knockouts of E. coli were performed using λ Red recombineering as described previously (52). Primers used to generate these deletions are listed in Table S2. To recover fatty acid auxotrophic mutants of E. coli, selections were performed on LB antibiotic plates supplemented with 0.001% oleic acid (18:1) and 0.005% NP-40 to increase solubility of the fatty acid. P1 transductions were used to move mutations into MG1655 following standard protocols (53).

### RNA isolation and quantitative reverse transcriptase PCR (qRT-PCR).

Total RNA was isolated from mid-log-phase cells grown using TRIzol (Invitrogen) following manufacturer guidelines. Crude RNA extracts were further purified and treated with DNase I (Zymo Research) using the RNA Clean and Concentrator kit (Zymo Research). For cDNA synthesis, the Superscript III first-strand synthesis kit (Invitrogen) was used following the manufacturer’s recommended protocols. Quantitative PCRs were performed using the Maxima Sybr green master mix (Thermo Scientific) and run on a Stratagene MX3000P qPCR system. For quantification of target transcripts, the *gyrB* gene (PBPRA0011) was used as an internal reference, and differences in expression were calculated using the threshold cycle (ΔΔ*C_T_*) method. Primers for qPCR experiments are listed in Table S2 in the supplemental material.

### Fatty acid analysis.

Late-log-phase cultures were harvested by centrifugation and cell pellets rinsed once with 50% sea salt (Sigma) solution (16 g/liter) and stored at −80°C. Cell pellets were lyophilized and fatty acids were converted to fatty acid methyl esters and analyzed by gas chromatography-mass spectrometry using previously described methods (1). Fatty acids were identified based on their retention times and mass spectra and compared to authentic standards when necessary.

### Isolation of suppressor strains.

Fatty acid auxotrophs were grown to late log phase aerobically in 2216 medium supplemented with 0.05% Tween 80 (18:1) at 15°C. Cells were harvested by centrifugation and washed three times with 2216 broth to remove the fatty acid supplement. Washed cells were inoculated into 2216 liquid medium, which was used to fill heat-sealed sterile polyethylene transfer bulbs (Samco), and incubated at 30 MPa in stainless steel pressure vessels as described previously (49). Growth was monitored visually by eye or by OD_600_ readings. Culture bulbs displaying high levels of growth were sterilely opened, and serial dilutions were plated on 2216 agar. Larger colonies were clonally isolated by restreaking onto 2216 agar plates, and individual colonies were screened for growth on agar with and without 0.05% Tween 80 (18:1). Growth on plates was assessed by eye, and colonies that grew equally well on both media were designated putative suppressor strains and saved for further analysis.

### Identification of suppressor mutations.

High-quality genomic DNA was isolated from suppressor mutants using a Wizard genomic DNA kit following manufacturer guidelines for Gram-negative bacteria. Genomic DNA was submitted to Core facilities at the University of California, Davis (UC Davis), for library construction. Genomic libraries were sequenced on a HiSeq4000 Illumina platform to generate paired-end or single-end 150-bp reads. Reads were quality checked by fastqc version 0.11.3 (54). Reads that passed the initial quality control were mapped to the published SS9 genome sequence (55) using breseq version 0.33.2 (29) using default parameters. PCR and sequencing of purified products validated mutations of interest.
