# Supplementary material for: Genetic Suppression of Lethal Mutations in Fatty Acid Biosynthesis Mediated by a Secondary Lipid Synthase
Source: Appl Environ Microbiol. 2021 May 26;87(12):e00035-21. doi: 10.1128/AEM.00035-21 (PMC8174602; doi:10.1128/AEM.00035-21)
Supplement: SUPPLEMENTAL FILE 2 — Supplemental material. Download aem.00035-21-s0002.pdf, PDF file, 1.1 MB [file aem.00035-21-s0002.pdf]

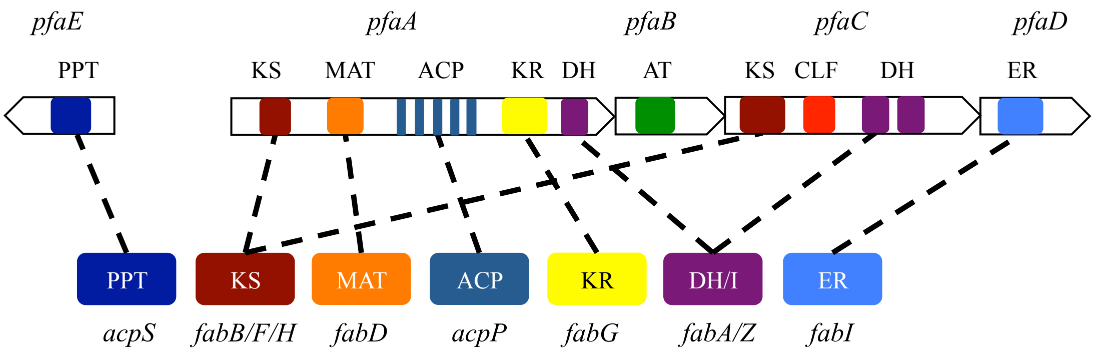

Supplemental Figure 1- Fatty acid biosynthesis pathways in *Photobacterium profundum* SS9. Comparison of the genes and functions comprising the Pfa synthase and their respective functional counterparts found in the Type II FAS. Domain designations include: phosphopantetheinyl transferase (PPT), β-ketoacyl synthase (KS), malonyl-CoA:ACP transacylase (MAT), acyl carrier protein (ACP), ketoacyl reductase (KR), dehydratase/ isomerase (DH), acyltransferase (AT), chain-length factor (CLF), enoyl reductase (ER).

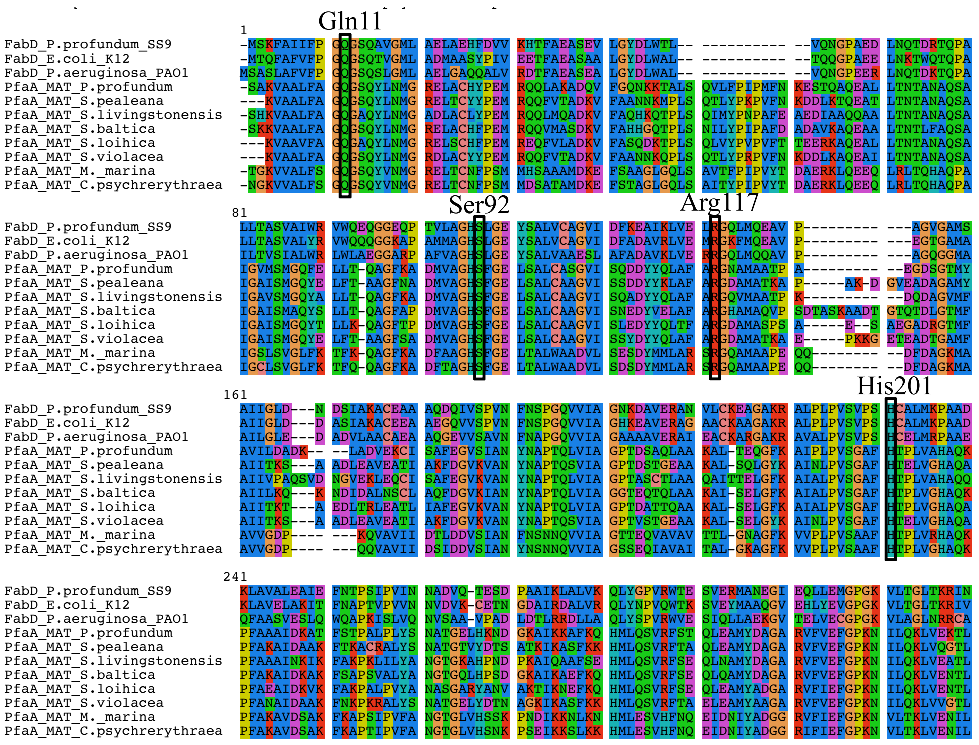

Supplemental Figure 2- Partial amino acid alignments of FabD and MAT domains from various strains. Previously characterized active site residues of FabD from *E.coli* are boxed including the active site Ser92. The *E.coli* FabD residues; Gln11, Arg117, His201 are involved in substrate recognition and catalysis of malonyl group transfer from CoA to ACP. These residues are universally conserved amongst FabD homologs and within the embedded MAT domains of various PfaA homologs.

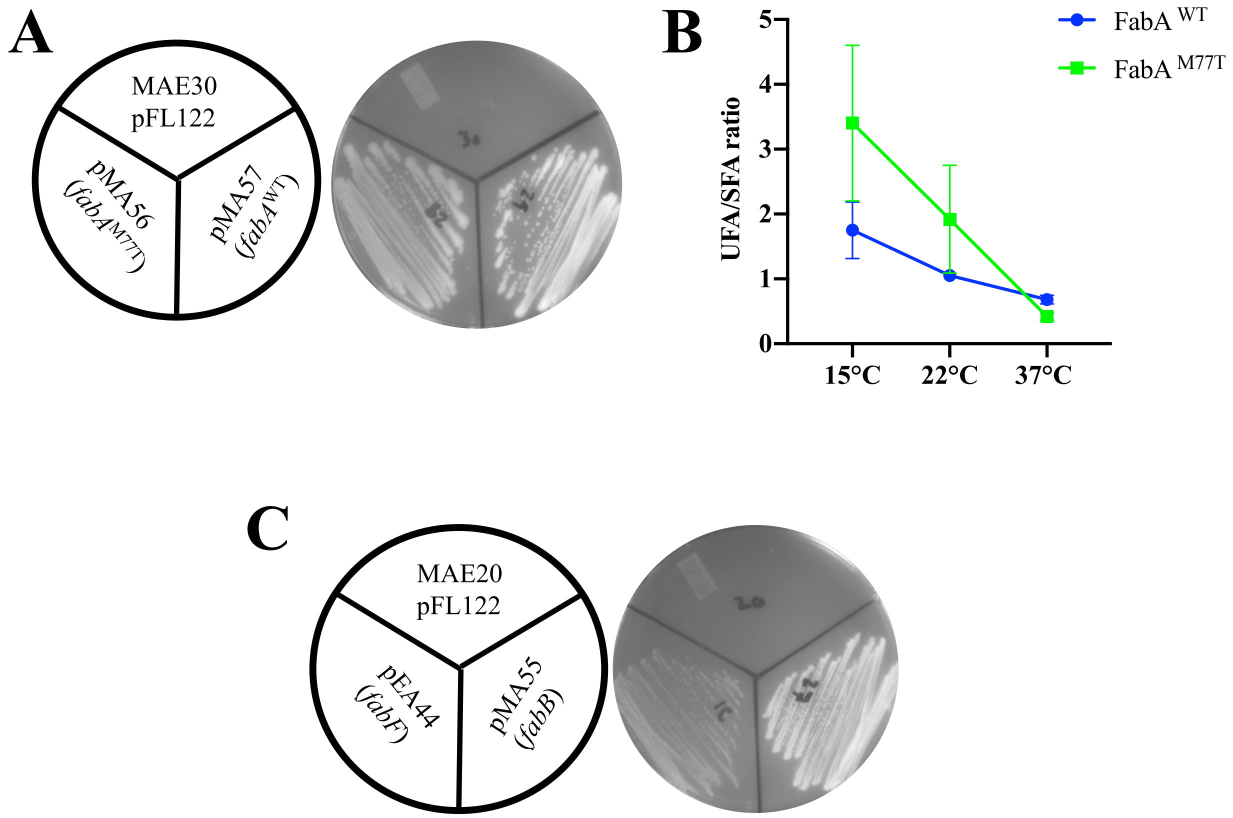

Supplemental Figure 3- Complementation of UFA auxotroph *E. coli* strains with Type II FAS genes from *P. profundum* SS9. (A) Both $fabA^{WT}$ and $fabA^{M77T}$ from *P. profundum* SS9 complement the Δ*fabA* mutant (MAE30). (B) Unsaturated to saturated fatty acid ratios (UFA/ SFA ratio) of MAE30 strain complemented by either $fabA^{WT}$ or $fabA^{M77T}$ variants. (C) Plasmids containing either *fabB* (pMA55) or *fabF* (pEA44) complement the Δ*fabB* mutant (MAE20). Respective *E. coli* strains were transformed with the indicated vectors and transformants recovered on LB Streptomycin plates supplemented with oleic acid. Individual transformants were restreaked onto plates without oleic acid and incubated at 37°C for 24hr before being imaged.

Supplemental Table S1 - Type II FAS biosynthetic homologs identified in *P. profundum* SS9.

Gene	Locus tag(s)	NCBI Accession No.	Notes
fabA	PBPRA1773	WP_011218488	Transcriptionally up-regulated at 30MPa (1)
fabB	PBPRA2658, PBPRB0107	WP_011219315, WP_011220206	Essential in *E.coli,* not essential in SS9, refer to text
fabD	PBPRA1194	WP_011217935	Essential in *E.coli,* not essential in SS9, refer to text
fabF	PBPRA1197, PBPRB0104	WP_011217938, WP_011220203	Complements *E.coli* Δ*fabB,* refer to text
fabG	PBPRA1195, PBPRB0105, PBPRB1106, PBPRB1562	WP_011217936, WP_011220204, WP_011221168, WP_011221582	Reduces β-Keto groups after ketosynthase reaction
fabH	PBPRA1193, PBPRA2001	WP_011217934, WP_011218703	Performs first condensation in Type II FAS (2)
fabV	PBPRA2423, PBPRA2527	WP_011219091, WP_011219192	Confers resistance to Triclosan in Vibrio sp. (3)
fabY	PBPRB1012	WP_011221079	Initiates Type II FAS in *Pseudomonas aeruginosa* PAO1 (4)
fabZ	PBPRA2957	WP_011219558	Dehydratase primarily involved in SFA production
fabR	PBPRA3467	WP_011219979	Regulates *fabA/B* in *E.coli* (5)
fadR	PBPRA2608	WP_011219266	Regulates *fabA/B* as in *E.coli,* (6, 7)
acpP	PBPRA1196	WP_011217937	Carries acyl groups in Type II FAS
acpS	PBPRA3085	WP_011219660	Cognate PPTase for AcpP
desA	PBPRB0742	WP_011220825	Presumably complements loss of *fabA/B* as seen in *P. aeruginosa* PAO1 (8)

Supplemental Table S2 - Primers used in this study.

Name	Sequence (5' -> 3')
fabB 5F	TACGATTCTAGAGTGATTAAACCCTCATCAAG
fabB 5R	GCCAGACGACCTAAGCCAGTCGGTTTATTCACAGAAAGTGG
fabB 3F	GACTGGCTTAGGTCGTCTGGCGGAAGTAAGGGGCTATACGT
fabB 3R	TACGATGAGCTCTGACAAACTCAGCATCAGCA
ΔfabB ver F	GTGCCTTCAGTGGTTTGATG
ΔfabB ver R	ATCACCATGACGGCATTAGG
fabA 5F	TGACTGGTACCTTGCCATTACGGGTGCAGTA
fabA 5R	GATCCGCGGCCGCGCTGGACCATAAAGTTCACC
fabA 3F	GATCCGCGGCCGCACAAACTTCTAAGTCGTCCC
fabA 3R	TATAGGAGCTCGGTTGAGGACGAACTATGAA
ΔfabA ver F	TGAGTGCCGCGCTAAACTTA
ΔfabA ver R	TGGTTCCGATCCCAGATGGT
desA 5F	TGACTGGTACCCTTAGAGGTGTATGCAGAGC
desA 5R	GACTGGCTTAGGTCGTCTGGCGGTGGTTTAGTGGTCGACAT
desA 3F	GCCAGACGACCTAAGCCAGTCCTTAGGCGGGATTTAACTCG
desA 3R	TATAGGAGCTCCAACCGCTGGCAATTCAATG
ΔdesA ver F	GTATGGAGCTTGAAGGTGCT
ΔdesA ver R	CAGCCAGAACTTACTCGCAT
pFL122 fabB F	TACGATGGATCCGTGATTAAACCCTCATCAAG
pFL122 fabB R	TACGATGTCGACTGACAAACTCAGCATCAGCA
pFL122 fabA F	ACGATCGAATTCCCTAACAGGCACTCAAGGTA
pFL122 fabA R	TATAGTCTAGAGGGACGACTTAGAAGTTTGT
pMUT100 fabD F	ACGATGGATCCGCAAGAACAAGGTGGTGAAC
pMUT100 fabD R	ACGATGTCGACTAATTGCAGCAGGATCGCTC
fabD 5F	TGACTGGTACCGCCTAGCAATACCTGATCAC
fabD 5R	GACTGGCTTAGGTCGTCTGGCGTTCACCACCTTGTTCTTGC
fabD 3F	GCCAGACGACCTAAGCCAGTCGAGCGATCCTGCTGCAATTA
fabD 3R	TATAGGAGCTCCACGGTAATACCACGAGATG
SS9 ΔfabD ver F	GTCGTTATTGGTGCAAGTGA
SS9 ΔfabD ver R	TTGCTAGTGTAGCAGCACGT
K12 fabD::kan F	GGGCTCCGCGCTGGTTCGTTTCTAGGATAAGGATTAAAACG TGTAGGCTGGAGCTGCTTC
K12 fabD::kan R	CCAGTGCGATTTTTCCTTCAAAATTCATGATTTTCCTCTTAT GGGAATTAGCCATGGTCC
K12 ΔfabD ver F	GGGCAGTTGGTTCTGCTTGA
K12 ΔfabD ver R	CGAGCGTTTCAGCAATTGCG
pBAD K12 fabD F	GCGCGAATTCATGACGCAATTTGCATTTGT
pBAD K12 fabD R	GCGCTCTAGACTTTTAAAGCTCGAGCGC

Supplemental Table S3- Fatty acid profile of MAP41 at 15°C.

Fatty acid	Mean % fatty acid[a] MAP41 (Δ*fabA*Δ*desA* *fabD*::pMA71)
12:0	3.90 ± 0.10
14:0	10.11 ± 0.07
14:1	0.00 ± 0.00
16:0iso	2.17 ± 0.39
16:0	43.26 ± 1.76
16:1	0.00 ± 0.00
12-OH	2.10 ± 0.17
18:0	0.57 ± 0.06
18:1	0.00 ± 0.00
20:5	37.15 ± 0.46
22:6	1.37 ± 0.12

[a] Data represent the average ± standard deviation of triplicate samples

Supplemental References

1. **Campanaro S**, **Vezzi A**, **Vitulo N**, **Lauro FM**, **D'Angelo M**, **Simonato F**, **Cestaro A**, **Malacrida G**, **Bertoloni G**, **Valle G**, **Bartlett DH**. 2005. Laterally transferred elements and high pressure adaptation in Photobacterium profundum strains. BMC Genomics **6**:122.
2. **Cronan JE**, **Rock CO**. 2008. Biosynthesis of Membrane Lipids. EcoSal Plus **3**.
3. **Massengo-Tiassé RP**, **Cronan JE**. 2008. Vibrio cholerae FabV defines a new class of enoyl-acyl carrier protein reductase. J Biol Chem **283**:1308–1316.
4. **Yuan Y**, **Leeds JA**, **Meredith TC**. 2012. Pseudomonas aeruginosa directly shunts β-oxidation degradation intermediates into de novo fatty acid biosynthesis. J Bacteriol **194**:5185–96.
5. **Feng Y**, **Cronan JE**. 2011. Complex binding of the FabR repressor of bacterial unsaturated fatty acid biosynthesis to its cognate promoters. Mol Microbiol **80**:195–218.
6. **Allemann MN**, **Allen EE**. 2020. Genetic Regulation of the Bacterial Omega-3 Polyunsaturated Fatty Acid Biosynthesis Pathway. J Bacteriol **202**.
7. **Cronan JE**, **Subrahmanyam S**. 1998. FadR, transcriptional co-ordination of metabolic expediency. Mol Microbiol **29**:937–43.
8. **Zhu K**, **Choi K-H**, **Schweizer HP**, **Rock CO**, **Zhang Y-M**. 2006. Two aerobic pathways for the formation of unsaturated fatty acids in Pseudomonas aeruginosa. Mol Microbiol **60**:260–73.
